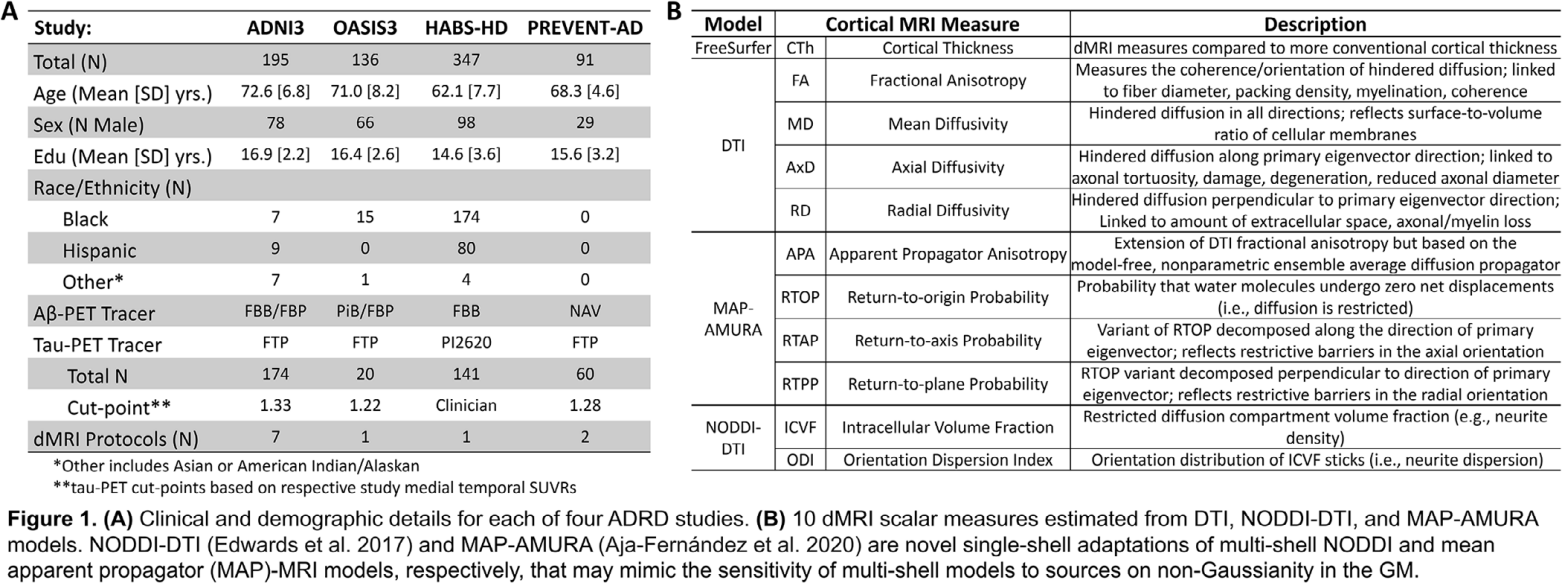# Cortical microstructure associations with amyloid PET in cognitively normal people

**DOI:** 10.1002/alz70856_098491

**Published:** 2025-12-24

**Authors:** Talia M Nir, Sunanda Somu, Kevin Low, Siddharth Narula, Julio E Villalon‐Reina, Emma J Gleave, Sophia I Thomopoulos, Noelle Lee, Meredith N. Braskie, Paul M. Thompson, Neda Jahanshad

**Affiliations:** ^1^ Imaging Genetics Center, Mark and Mary Stevens Neuroimaging & Informatics Institute, University of Southern California, Marina del Rey, CA, USA; ^2^ Imaging Genetics Center, Mark and Mary Stevens Neuroimaging and Informatics Institute, Keck School of Medicine, University of Southern California, Marina del Rey, CA, USA

## Abstract

**Background:**

Diffusion MRI (dMRI) is sensitive to small changes in brain microstructure and may offer sensitivity to early Alzheimer's disease (AD) neuropathology that precedes macrostructural brain changes. Subtle Aβ effects may be better captured by dMRI measures in cortical gray matter (GM), where early AD histopathological changes occur, compared to more conventional cortical thickness (CTh) measures. Here, we evaluated relationships between Aβ‐PET and single‐shell cortical dMRI measures in cognitively normal (CN) individuals from four AD studies. For comparison, CTh was also evaluated.

**Methods:**

T1w, dMRI, and Aβ‐PET data were analyzed in 769 CN participants from ADNI3, HABS‐HD, OASIS3, and PREVENT‐AD (Figure 1a); 425 participants had tau‐PET data. In addition to DTI, advanced single‐shell NODDI‐DTI and MAP‐AMURA models were fit to dMRI data. CTh and 10 mean dMRI measures (defined in Figure 1b) were extracted from 34 cortical regions parcellated from T1w images with FreeSurfer, and the full cortex. Random‐effects linear regressions were used to test for associations between regional cortical MRI measures and Aβ‐PET centiloids (CL), adjusting for age, sex, education, ethnicity/race, total CTh (excluded from CTh analyses), intracranial volume, and grouping by study dMRI protocol. We also tested the interactive effects of Aβ‐CL and tau‐PET positivity on significant cortical measures; tau positivity was defined for each respective study (Figure 1a).

**Results:**

Aβ‐CL was associated with three dMRI measures and CTh (P_FDR_<0.05; Figure 2a). Limited regional associations were found between greater Aβ and higher DTI FA and CTh. More widespread associations were detected with advanced dMRI models; greater Aβ was associated with higher APA and lower ODI in the full cortex. ODI was moderated by tau; tau+ individuals showed steeper negative ODI slopes with respect to Aβ burden (Figure 2b).

**Conclusions:**

Compared to CTh, advanced dMRI measures showed more widespread associations with Aβ load. Greater hindered diffusion (i.e., higher APA, FA) associations with greater Aβ burden may reflect cellular hypertrophy or inflammatory microglia infiltration increasing the number of diffusion barriers in early stages, irrespective of tau status. In contrast, lower neurite dispersion (ODI) could be driven by neurite loss and neurodegeneration; these effects were greater in tau+ participants. dMRI measures may offer insight into early pathological disease processes.